# What types of tobacco control public service advertisements work for Chinese adolescents? A mixed-methods study

**DOI:** 10.18332/tid/211650

**Published:** 2025-11-04

**Authors:** Yu Chen, Haoyi Liu, Shiyu Liu, Yujiang Cai, Jing Xu, Xinrui Yang, Kin-Sun Chan

**Affiliations:** 1 School of Art and Communication, Fujian Polytechnic Normal University, Fuqing, China; 2 Department of Media and Communication, City University of Hong Kong, Hong Kong SAR, China; 3 School of Public Health, Xi’an Jiaotong University, Xi’an, China; 4 School of International Studies, Peking University, Beijing, China; 5 School of Journalism and Communication, Peking University, Beijing, China; 6 Institute for Global Health and Development, Peking University, Beijing, China; 7 Faculty of Social Sciences, University of Macau, Macao SAR, China

**Keywords:** tobacco control, public service advertisements, adolescents, health communication, mixed-methods research

## Abstract

**INTRODUCTION:**

Adolescent tobacco use has become a serious global public health problem, and effective tobacco control public service advertisements (PSAs) are crucial for reducing adolescent smoking rates. The study aims to employ a mixed-methods approach combining quantitative surveys and qualitative focus groups to evaluate the effectiveness of different types of tobacco control PSAs among Chinese adolescents, identify effective advertising characteristics and content elements, and provide empirical evidence for optimizing youth tobacco control communication strategies.

**METHODS:**

A total of 125 students aged 10–18 years were recruited from six primary and secondary schools in Beijing and Kunming from November 2020 to April 2021. Participants completed Likert-scale ratings measuring advertisement effectiveness after viewing eight tobacco control PSAs and participated in focus group interviews. Quantitative data were analyzed using independent samples t-tests, Spearman correlation analysis, and multivariable logistic regression analysis, while qualitative data were analyzed using thematic analysis. All statistical tests were two-tailed with significance set at p<0.05.

**RESULTS:**

Quantitative analysis revealed that PSAs employing ‘testimonials’ and ‘disease’ frameworks were most strongly associated with prevention intentions, while those using ‘celebrity endorsement’, ‘humor’ and ‘appearance damage’ frameworks showed the weakest associations. Kunming adolescents showed significantly higher advertisement acceptance scores than Beijing adolescents (mean difference=0.21; 95% CI: 0.04–0.38, p<0.05). The 10-item effectiveness scale demonstrated good internal consistency (Cronbach’s α=0.82). Qualitative analysis identified effective characteristics including presentation of specific health hazards, use of testimonials, and fear appeals; ineffective characteristics included non-specific harm presentation, use of humorous elements, and appearance damage content.

**CONCLUSIONS:**

Tobacco control PSA design should consider strategies combining disease warnings with real-life testimonials, avoid humorous advertisements and industry-sponsored messaging, and consider regional cultural differences. Distribution through online and social media platforms frequently used by adolescents may enhance reach. Future longitudinal research with broader geographical sampling is needed to confirm these findings.

## INTRODUCTION

China has over 340 million smokers, with more than one million deaths annually from tobacco-related diseases^1,2^. Adolescence, as a critical period of physical and mental development, is also a high-risk period for smoking initiation^3^. Survey data from 2023 showed that the current smoking rate among Chinese secondary school students was 4.2%, with approximately 3.76 million student smokers^4^, indicating that adolescent tobacco use has become a major public health problem requiring urgent intervention in China.

Extensive research has confirmed that antismoking advertising campaigns can effectively prevent adolescent smoking and reduce adolescent smoking rates^5^. Three major international tobacco control campaigns provide important references for understanding effective advertising elements. The Centers for Disease Control and Prevention (CDC’s) *Tips from Former Smokers*® (TIPS) campaign has set the gold standard for evidence-based tobacco control advertising. Since its launch in 2012, the campaign has generated 16.4 million quit attempts and over one million sustained quits, with remarkable cost-effectiveness at $3800 per early death prevented^6^. The success of the TIPS campaign stems from featuring real people living with smoking-related diseases rather than actors, focusing on the difficulty of living with chronic conditions rather than death messaging^7^.

The US Truth Campaign, launched in 2000, has prevented 450000 adolescent smoking initiations through anti-industry messaging strategies, achieving $1818 per quality-adjusted life year saved^8^. The campaign employed anti-corporate manipulation messaging, positioning tobacco control as a youthled social movement, showing particular effectiveness among high sensation-seeking adolescents^9^. The FDA’s ‘The Real Cost Campaign’, launched in 2014, prevented 348000–587000 youth smoking initiations by emphasizing personal costs of smoking, achieving $1337 per quality-adjusted life year saved^10^. The campaign employed loss-frame messaging focusing on smoking’s impact on appearance, loss of control, and addiction, demonstrating broad applicability across diverse demographic groups^11^.

In contrast, tobacco industry-sponsored youth prevention campaigns have been proven counterproductive for tobacco control. Philip Morris’s ‘Think. Don’t Smoke’ campaign, launched in 1998, serves as a cautionary example of industry manipulation tactics. Academic research found that exposure to Philip Morris ads was associated with more favorable attitudes toward the tobacco industry and increased openness to smoking among adolescents^12^. A randomized controlled trial with 832 high school students revealed that industry ads ‘engendered more favorable attitudes toward tobacco companies’ with no reduction in smoking intentions^13^.

In China’s distinctive context, the State Tobacco Monopoly Administration (STMA) paradoxically participates in the WHO Framework Convention on Tobacco Control action group while simultaneously overseeing the world’s largest tobacco market^14^. China’s tobacco control landscape presents unique challenges, ranking in the bottom 20% globally for WHO Framework Convention on Tobacco Control (FCTC) compliance, with fundamental conflicts of interest undermining tobacco control implementation^14^. The State Tobacco Monopoly Administration (STMA) and China National Tobacco Corporation (CNTC) are essentially the same organization, creating an unprecedented situation where the tobacco industry regulates itself ^15^. This fundamental conflict of interest creates unprecedented scenarios where tobacco industry entities might develop youth-targeted ‘control’ campaigns that appear beneficial but ultimately serve corporate interests rather than public health objectives. Understanding adolescent responses to industry-sponsored messaging becomes particularly crucial for Chinese policymakers and health advocates who must distinguish between authentic tobacco control efforts and sophisticated public relations campaigns designed to enhance industry credibility while maintaining market access.

Despite the wealth of international research on tobacco control advertisement effectiveness, significant gaps remain in understanding how different message characteristics resonate with adolescents across diverse cultural and policy contexts. While studies have established the general effectiveness of fear appeals, testimonial messaging, and graphic health warnings^16,17^, limited research has systematically compared the relative effectiveness of different advertisement frameworks among Chinese adolescents within varying regional tobacco control environments. This gap is particularly important given China’s complex policy landscape where regional variations in tobacco control implementation may significantly influence adolescent responses to health messaging^18^. Furthermore, while industry-sponsored advertisements have been studied extensively in Western contexts^12^, their potential impact within China’s unique regulatory environment where tobacco companies participate in policy development remains unexplored.

This study addresses these research gaps by systematically evaluating eight tobacco control PSAs representing diverse message frameworks, appeal types, and source credibility among Chinese adolescents in two cities with distinctly different tobacco control policy environments. The research aims to identify effective advertising characteristics and content elements while providing empirical evidence for optimizing youth tobacco control communication strategies in China’s complex regulatory landscape.

## METHODS

### Study design

This study employed a concurrent mixed-methods design, combining cross-sectional quantitative surveys with qualitative focus group interviews^19^. This methodological approach was chosen to achieve triangulation, with quantitative advertisement rating results complementing and corroborating specific feedback from adolescents in interviews to identify effective advertising formats and content characteristics^20^.

The mixed-methods procedure followed a systematic sequential approach within each data collection session. Participants first individually viewed all eight tobacco control PSAs in randomized order and completed quantitative evaluations for each advertisement using structured questionnaires. Following completion of the quantitative assessment phase, participants were organized into focus groups of 10–12 individuals stratified by education level and gender. Trained facilitators with experience in adolescent health research conducted semi-structured interviews using a standardized guide to minimize interviewer bias to explore the underlying reasons for advertisement effectiveness ratings, ensuring all participants had opportunities to contribute their perspectives on what made specific advertisements compelling or ineffective for tobacco prevention. This sequential approach ensured that qualitative insights could build upon and contextualize quantitative findings while avoiding potential bias from group discussions influencing individual advertisement ratings.

### Participants

This study employed purposive sampling, conducted from November 2020 to April 2021 in two primary schools, two middle schools, and two high schools in Beijing and Kunming. Schools were purposively selected based on: 1) representation of different socioeconomic areas within each city, 2) willingness to participate, and 3) absence of recent tobacco control interventions. Within schools, classes were randomly selected, and all students meeting inclusion criteria were invited to participate. These two cities were selected to maximize sample diversity and enable comparison. Beijing, located in northern China, has performed relatively well in tobacco control work and has implemented smoke-free regulations compliant with the WHO Framework Convention on Tobacco Control, with relatively high public tobacco control awareness^21^ Kunming, located in southern China and the capital of Yunnan Province, represents a region where the local economy is highly dependent on tobacco, as Yunnan is a major tobacco production and consumption province with relatively low public tobacco control awareness^22^.

A total of 125 participants were recruited, divided into 12 groups by education level and gender: primary school boys, primary school girls, middle school boys, middle school girls, high school boys, and high school girls, with 10–12 participants per group. Inclusion criteria included: 1) residing in Kunming or Beijing, 2) aged between 10–18 years, 3) having adequate comprehension abilities (able to understand Mandarin Chinese and complete age-appropriate questionnaires independently), and 4) parent agreeing to participate in testing and signing informed consent. School administrators distributed information sheets to parents two weeks before data collection. Parents provided written consent, and students provided assent on the day of data collection. The participation rate was 89% of eligible students.

### Test materials

The study selected eight tobacco control PSAs of varying durations (seconds) with different narrative frameworks and appeal approaches (Table 1). These advertisements included materials from the US TIPS campaign^6^, Truth Campaign^8^, and ‘The Real Cost’ campaign^11^, as well as locally produced Chinese advertisements, including the ‘Think. Don’t Smoke’ advertisement produced by Philip Morris tobacco company, providing a unique opportunity to evaluate the impact of industry-sponsored advertising in China. All PSAs were publicly available health communication materials. Usage for research purposes falls under fair use provisions for educational and scientific research. PSAs are archived and available from the corresponding author upon request for research purposes.

**Table 1:** Description of tobacco control public service advertisements (PSAs) used in mixed-methods study, Beijing and Kunming, China, November 2020–April 2021 (N=125)

*No.*	*PSA*	*Duration* *(seconds)*	*Type*	*Framework*	*Appeal*	*Description*
1	‘Suffering from emphysema’	30	Victim testimonial	Disease	Emotional	Middle-aged man with emphysema, heavy breathing and coughing
2	‘A tip from a former smoker’	26	Victim testimonial	Real testimonial	Emotional + rational	51-year-old woman with throat cancer
3	‘Cigarettes are eating you alive’	25	Harm description	Disease + death	Emotional + rational	Visual demonstration of smoking damage to organs
4	‘Smoke-free generation’	34	Celebrity endorsement	Celebrity teaching	Rational	Celebrity encouraging people not to smoke
5	‘Smoking, nicotine and addiction’ (the real cost)	39	Nicotine addiction effects	Disease	Rational	Animation showing nicotine addiction effects
6	‘The makeover you never wanted’ (the real cost )	18	Appearance impact	Appearance damage	Emotional	Young woman discovering aging effects from smoking
7	‘Cold truth’	38	Comprehensive effects	Disease	Rational	Youth-focused narration about smoking consequences
8	‘Think, don’t smoke’	30	Industry ‘control’ ad	Humor	Emotional	Party scene with humorous elements

Type refers to the message delivery approach. Framework indicates the primary health message strategy. Appeal denotes the persuasive approach (emotional, rational, or combined). Description provides key visual and narrative elements.

### Survey instrument

The questionnaire consisted of two main parts. The first part collected basic demographic information including gender, age, media usage, and smoking status. The second part featured Likert scales designed for the eight tobacco control PSAs, evaluating advertisement effectiveness across cognitive-attitudinal-behavioral dimensions using 5-point Likert scales (1=strongly disagree, 5=strongly agree). The effectiveness measures were adapted from validated instruments used in international tobacco control research^18,23^, including assessments of perceived credibility, emotional impact, behavioral intentions, and sharing likelihood. The effectiveness scale demonstrated good internal consistency in our sample (Cronbach’s α=0.82). Higher scores on ‘makes uncomfortable’ indicate greater emotional impact, which tobacco control literature suggests enhances message effectiveness through fear appeal mechanisms.

### Data collection process

Data collection occurred in school computer labs or classrooms with projection equipment. Students viewed each PSA individually on screens, immediately completing ratings before viewing the next advertisement in randomized order. This ensured independent ratings without peer influence. Questionnaires used anonymous ID codes. Data collection occurred in supervised settings without teachers present to minimize coercion. Completed forms were sealed in envelopes by students themselves to ensure privacy and confidentiality.

### Data analysis

Quantitative data analysis was conducted using SPSS 27.0. Descriptive statistics included frequencies and percentages, and means and standard deviations. Independent samples t-tests were used for regional comparisons, Spearman correlation examined associations between variables, and multivariable logistic regression with robust standard errors identified predictors of advertisement effectiveness while accounting for potential within-person correlation. Likert scale responses 1–3 were combined as ‘neutral or negative’ and 4–5 as ‘positive’, with scores expressed as positive response probabilities^24^. All statistical tests were two-tailed with significance set at p<0.05. Given the exploratory nature of this study and to avoid Type II errors in identifying potentially effective advertisement characteristics, we did not apply corrections for multiple comparisons. This limitation is acknowledged and key findings should be confirmed in future studies. Qualitative data were managed using Nvivo 12.0 software and analyzed using thematic analysis methods^23^. Triangulation was achieved by comparing quantitative effectiveness ratings with qualitative explanations of advertisement appeal characteristics, ensuring convergent validation of findings across methodological approaches. We tested for PSA×city interactions but found none significant (all p>0.10), suggesting consistent patterns across cities.

### Ethics review

This study was approved by the Peking University Biomedical Ethics Committee (PU IRB), with ethics review number IRB00001052-20056, which covered both study sites through institutional agreements.

## RESULTS

### Participant characteristics

A total of 125 participants were recruited from six schools, with males comprising 49.6% (n=62) and females 50.4% (n=63), showing balanced gender distribution. Regarding media usage, television (85.6%) and internet (81.6%) were the two primary information channels for adolescents. Over half (53.6%) of participants lived with smokers at home; smoking rates among surveyed adolescents were low at only 1.6% (Table 2).

**Table 2:** Participant characteristics by region in mixed-methods study of tobacco control PSAs, Beijing and Kunming, China, November 2020–April 2021 (N=125)

*Characteristics*	*Category*	*Total* *n (%)*	*Beijing* *n (%)*	*Kunming* *n (%)*
**Gender**	Male	62 (49.6)	32 (47.8)	30 (51.7)
	Female	63 (50.4)	35 (52.2)	28 (48.3)
**Education level**	Primary (10–12 years)	35 (28.0)	18 (26.9)	17 (29.3)
	Middle (13–15 years)	41 (32.8)	22 (32.8)	19 (32.8)
	High (16–18 years)	49 (39.2)	27 (40.3)	22 (37.9)
**Media use**	Television	107 (85.6)	58 (86.6)	49 (84.5)
	Internet	102 (81.6)	54 (80.6)	48 (82.8)
	School platforms	53 (42.4)	29 (43.3)	24 (41.4)
**Living with smokers**	Yes	67 (53.6)	34 (50.7)	33 (56.9)
	No	58 (46.4)	33 (49.3)	25 (43.1)
**Smoking status**	Non-smoker	123 (98.4)	66 (98.5)	57 (98.3)
	Smoker	2 (1.6)	1 (1.5)	1 (1.7)

### Advertisement effectiveness ratings

The top three most effective advertisements for preventing adolescent smoking initiation were: ‘A tip from a former smoker’ (31.2%), ‘Cigarettes are eating you alive’ (24%), and ‘Suffering from emphysema’ (18.4%) (Table 3).

**Table 3:** Regional ranking of tobacco control public service advertisement (PSA) effectiveness in mixedmethods study, Beijing and Kunming, China, November 2020–April 2021 (N=125)

*Advertisement*	*Most effective prevention*			*Least effective prevention*		
	*Total* *n (%)*	*Beijing* *n (%)*	*Kunming* *n (%)*	*Total* *n (%)*	*Beijing* *n (%)*	*Kunming* *n (%)*
Suffering from emphysema	23 (18.4)	11 (16.4)	12 (20.7)	5 (4.0)	3 (4.5)	2 (3.4)
A tip from a former smoker	39 (31.2)	19 (28.3)	20 (34.5)	2 (1.6)	2 (3.0)	0 (0)
Cigarettes are eating you alive	30 (24.0)	17 (25.4)	13 (22.4)	1 (0.8)	1 (1.5)	0 (0)
Smoke-free generation	5 (4.0)	3 (4.5)	2 (3.4)	47 (37.6)	31 (46.0)	16 (27.6)
Smoking, nicotine and addiction	15 (12.0)	9 (13.4)	6 (10.3)	6 (4.8)	4 (6.0)	2 (3.4)
The makeover you never wanted	0 (0)	0 (0)	0 (0)	25 (20.0)	11 (16.4)	14 (24.1)
Cold truth	6 (4.8)	4 (6.0)	2 (3.4)	5 (4.0)	2 (3.0)	3 (5.2)
Think, don’t smoke	7 (5.6)	4 (6.0)	3 (5.1)	34 (27.2)	13 (19.4)	21 (36.2)

The three least effective PSAs for preventing smoking initiation were: ‘Smoke-free generation’ (37.6%), ‘Think, don’t smoke’ (27.2%), and ‘The makeover you never wanted’ (20%). These three advertisements represented respectively: celebrity endorsement, tobacco company ‘control’ advertising, and smoking’s impact on adolescent appearance.

### Detailed effectiveness indicator scores

Analysis of the ten effectiveness measurement indicators revealed consistent patterns across different advertisement types. The highest-rated advertisements scored particularly high on credibility, teaching new information, emotional impact, and behavioral intention measures, while the lowest rated advertisements showed poor performance across all indicators (ANOVA comparing high vs low effectiveness groups: F=15.3, p<0.001) (Table 4).

**Table 4:** Tobacco control PSA ten effectiveness measurement indicator scores in mixed-methods study, Beijing and Kunming, China, November 2020–April 2021 (N=125)

*Advertisement*	*Easy to* *understand*	*Teaches new* *things*	*Credible*	*Makes* *uncomfortable*	*Relevant to* *me*	*Concerns* *about* *secondhand* *smoke*	*Makes me* *think*	*Less likely to* *try smoking*	*Can effectively* *prevent* *smoking*	*Would share/discuss*
Suffering from emphysema	108 (86.4)	87 (69.6)	**111 (88.8)**	**86 (68.8)**	29 (23.2)	69 (55.2)	**88 (70.4)**	**110 (88.0)**	**112 (89.6)**	77 (61.6)
A tip from a former smoker	100 (80.0)	84 (67.2)	107 (85.6)	**80 (64.0)**	24 (19.2)	**75 (60.0)**	**88 (70.4)**	**114 (91.2)**	**113 (90.4)**	**85 (68.0)**
Cigarettes are eating you alive	**118 (94.4)**	**100 (80.0)**	**116 (92.8)**	**86 (68.8)**	43 (34.4)	**85 (68.0)**	**93 (74.4)**	**116 (92.8)**	**116 (92.8)**	79 (63.2)
Smoke-free generation	108 (86.4)	71 (56.8)	97 (77.6)	16 (12.8)	**58 (46.4)**	50 (40.0)	56 (44.8)	95 (76.0)	89 (71.2)	71 (56.8)
Smoking, nicotine and addiction	**119 (95.2)**	**110 (88.0)**	**117 (93.6)**	19 (15.2)	**77 (61.6)**	**73 (58.4)**	86 (68.8)	108 (86.4)	104 (83.2)	**80 (64.0)**
The makeover you never wanted	83 (66.4)	73 (58.4)	106 (84.8)	37 (29.6)	52 (41.6)	70 (56.0)	69 (55.2)	97 (77.6)	98 (78.4)	67 (53.6)
Cold truth	**119 (95.2)**	**99 (79.2)**	108 (86.4)	22 (17.6)	**57 (45.6)**	72 (57.6)	82 (65.6)	107 (85.6)	103 (82.4)	**93 (74.4)**
Think, don’t smoke	103 (82.4)	77 (61.6)	85 (68.0)	23 (18.4)	51 (40.8)	65 (52.0)	65 (52.0)	103 (82.4)	92 (73.6)	80 (64.0)

Data are given as n (%). Bold numbers indicate the top three scores for each indicator.

The effectiveness ranking advertisements (‘A tip from a former smoker’, ‘Cigarettes are eating you alive’, and ‘Suffering from emphysema’) consistently scored highest across multiple indicators, particularly for credibility, emotional impact (‘makes uncomfortable’), and behavioral intention measures (‘less likely to try smoking’ and ‘can effectively prevent smoking’). These advertisements achieved the optimal combination of high comprehensibility with strong emotional and behavioral responses.

### Regional difference analysis

Comparison between Beijing and Kunming revealed that the top three most effective advertisements for preventing smoking initiation were consistent across both cities. However, rankings differed for the least effective advertisements. In Beijing, the highest proportion (46%) considered the ‘Smoke-free generation’ least effective; in Kunming, the highest proportion (36.2%) considered ‘Think, don’t smoke’ least effective (Table 5).

**Table 5:** Regional and demographic factors associated with advertisement acceptance from multivariable logistic regression analysis, Beijing and Kunming, China, November 2020–April 2021 (N=125)

*Factor*	*Group*	*n*	*Mean score*	*β*	*T/r*	*95% CI*	*p*
**Region**	Kunming	58	3.96 ± 0.52	0.21	2.403	0.04–0.38	0.018*
	Beijing	67	3.75 ± 0.48				
**Gender**	Male	62	3.93 ± 0.47	0.16	1.871	-0.01–0.33	0.064
	Female	63	3.77 ± 0.54				
**Age**	-	-	-	-0.02	-0.042	-0.08–0.04	0.644
**Education level**	-	-	-	-0.01	-0.011	-0.12–0.10	0.901

Model adjusted for all variables shown. Robust standard errors used.

* p<0.05.

Independent samples t-test showed that the difference test between region and advertisement acceptance effectiveness had a p<0.05, indicating statistically significant differences in advertisement acceptance effectiveness between Beijing and Kunming students. Kunming students showed higher advertisement acceptance effectiveness compared to Beijing students (mean ± SD: 3.96 ± 0.52 vs 3.75 ± 0.48, mean difference=0.21, 95% CI: 0.04–0.38, p=0.018).

### Qualitative findings

Qualitative analysis identified characteristics of effective and ineffective smoking prevention videos from two aspects: ‘theme and information characteristics’ and ‘video characteristics and style’. Effective characteristics included: presentation of specific health hazards, use of real-life cases, positive perceived effectiveness, fear appeals, and detailed visual effects. Ineffective characteristics included: non-specific harm presentation, humorous elements, appearance damage content, low information acceptance, romantic content, bland presentation, and insufficient duration. Representative quotes for effective characteristics included:

*‘Because it’s a real person, it can be more moving, more warning about the dangers of smoking, and make people not want to smoke.’* (Student 8, high school male focus group, Kunming)

*‘I think that girl with a hole in her throat* … *is most thought-provoking.’* (Student 4, primary school male focus group, Beijing)

while representative quotes for ineffective characteristics included:

*‘It didn’t show the harm of smoking very well* … *this kind of presentation.’* (Student 6, middle school male focus group, Kunming)

*‘Plus most of it contains humorous elements, which might make people mistake it for a comedy.’* (Student 3, middle school female focus group, Beijing)

## DISCUSSION

### Advantages of testimonial and disease frameworks

This study found that tobacco control advertisements designed for adults showed strong associations with prevention intentions among adolescent populations. The top three effective advertisements: ‘A tip from a former smoker’, ‘Cigarettes are eating you alive’, and ‘Suffering from emphysema’, were all adult-targeted tobacco control materials, yet advertisements using real-life testimonials and disease frameworks were highly rated among adolescents. This finding aligns with international research, as the success of the TIPS campaign demonstrates that adult-oriented authentic testimonial campaigns are associated with influencing youth as a secondary audience^7,11,25^.

This result provides new empirical support for health communication theory, strengthening the application of ‘narrative persuasion theory’ in tobacco control^26^. Through vivid, authentic storytelling, audiences’ perceptions and attitudes toward smoking harm may be influenced^27^. For tobacco control communication practice, future advertisement creation should focus on discovering more authentic and representative cases, deeply presenting disease consequences caused by smoking.

Unlike Western studies showing limited effectiveness of fear appeals alone, Chinese adolescent sample responded positively to PSAs combining fear appeals with specific health information, suggesting cultural differences in message reception. Our findings both align with and diverge from Western tobacco control research. Consistent with international studies, testimonial-based advertisements showed strong associations with prevention intentions. However, our Chinese adolescent sample showed higher receptivity to authority-based messages and combined fear– health information appeals compared to anti-industry messaging effectiveness reported in US studies. This suggests cultural specificity in message reception that should inform campaign adaptation across different contexts.

### Validation through response indicators

The comprehensive response indicator analysis provides robust validation of the main findings through detailed measurement across ten validated dimensions. The stronger performance of testimonial and disease-focused advertisements becomes particularly evident when examining specific indicators such as credibility, emotional impact, and behavioral intentions. The top-performing advertisements achieved high credibility score, emotional impact (‘makes uncomfortable’) and behavioral intention scores (‘less likely to try smoking’), demonstrating the powerful combination of trustworthiness and motivational appeal that characterizes impactful tobacco control messaging.

These findings align with established frameworks in tobacco control advertisement research, where credibility and emotional engagement serve as primary pathways to behavior change^13,18,28^. The convergence of high scores across multiple indicators for the same advertisements provides strong evidence for the validity of both participant selection criteria and the underlying response constructs being measured.

### Triangulation of quantitative and qualitative evidence

The integration of quantitative effectiveness ratings with qualitative explanatory insights achieved robust triangulation that strengthens confidence in the research findings. Quantitative data revealed clear patterns of advertisement effectiveness, with testimonial and disease-focused advertisements ranking highest across multiple outcome measures. Qualitative analysis provided convergent validation by identifying the specific characteristics that adolescents found compelling in these same advertisements, including authentic personal stories, graphic health consequences, and emotional resonance.

This triangulation was particularly valuable in understanding why certain advertisements failed to achieve effectiveness. The quantitative data showed poor performance for celebrity endorsements and industry-sponsored advertisements, while qualitative insights revealed the underlying mechanisms, including perceptions of inauthenticity, lack of credible health information, and inappropriate humorous tone. The convergence of quantitative rankings with qualitative explanations provides robust evidence that the identified effective characteristics represent genuine drivers of adolescent response rather than measurement artifacts.

### Limitations of tobacco industry advertising

The ineffective characteristics of romance and humor mentioned by participants in the study both came from the tobacco company’s ‘Think, don’t smoke’ control advertisement. This finding is consistent with existing research conclusions that tobacco company control advertisements are not associated with prevention for adolescents^12,29^. Industry documents reveal these campaigns were primarily designed as public relations tools rather than genuine prevention efforts, with ‘Project Sunrise’ (1995–2006) explicitly aiming to rehabilitate Philip Morris’s image while deflecting regulatory pressure^12^.

These advertisements avoided mentioning health consequences and were never evaluated for actual youth smoking reduction. Instead, companies tracked ‘media hits’ and corporate image impacts. The campaigns’ directive ‘just say no’ messaging style triggers psychological reactance in adolescents, while portraying smoking as a ‘grown-up, sophisticated product’ potentially appeals to youth seeking maturity^13^. The Philip Morris advertisement ranked among the least effective in our study, consistent with research showing industry-sponsored youth prevention campaigns may actually normalize smoking by presenting it as an adult choice rather than a health hazard.

### Regional policy contexts and urban tobacco control implementation

The regional differences in tobacco control PSA feedback between Beijing and Kunming adolescents reveal important considerations for tobacco control campaign design in China’s diverse policy landscape. Both Beijing and Kunming are major urban centers, yet they represent distinctly different tobacco control policy environments that significantly influence adolescent responses to health messaging. These findings contribute to understanding appropriate youth-targeted tobacco control campaigns across China’s varied regional contexts.

Beijing represents a progressive tobacco control environment where comprehensive smoke-free policies have been successfully implemented through strong municipal leadership and effective enforcement mechanisms^30^. The city’s adolescents demonstrate relatively mature tobacco control awareness, having been extensively exposed to various forms of antismoking messaging through multiple channels. This prolonged exposure may create a form of ‘message saturation’ where conventional tobacco control advertisements lose their impact due to familiarity^31^, potentially explaining why Beijing adolescents showed lower overall advertisement acceptance scores.

In contrast, Kunming operates within a fundamentally different policy context where tobacco economic interests remain highly influential in local governance and public discourse^23^. As the capital of Yunnan Province, China’s leading tobacco production region, Kunming adolescents exist within a social environment where tobacco industry presence is more normalized and tobacco control messaging is less prevalent^23^. Under these circumstances, tobacco control PSAs function as relatively novel information sources, potentially explaining the significantly higher advertisement acceptance scores observed among Kunming participants.

### Communication strategy implications for the digital age

This study found that television and internet are the two major channels for adolescents to obtain information. This indicates that both need to be considered in tobacco control campaign practice^32^. On the one hand, the wide coverage of television can be utilized to broadcast impactful tobacco control advertisements during prime time. On the other hand, customized tobacco control content should be developed according to the characteristics of different websites and social media platforms.

Recent research shows that exposure to tobacco brand content on social media is associated with adolescent tobacco use initiation. Research found that youth with no prior tobacco use who engaged with tobacco brand content on social media had higher likelihood of tobacco initiation and increased risk for multi-product use. This makes authentic health messaging increasingly crucial^11^.

### Limitations

This study has certain limitations. The research sample was limited to school students in Beijing and Kunming, which may not fully represent adolescent populations in other regions of China. Differences in economic development levels, cultural backgrounds, and tobacco control policy environments across regions may affect the generalizability of results. The study primarily relied on self-reported data, which may be subject to social desirability bias and potential misclassification, particularly on sensitive topics involving smoking behavior. The cross-sectional design limits the ability to infer causality and cannot determine the temporal relationship between advertisement exposure and attitude changes. The purposive sampling may limit generalizability to rural or lower resource settings and may overrepresent motivated students whose responses differ from the general adolescent population.

Additional limitations include potential residual confounding despite multivariable adjustment, lack of fully adjusted models for all analyses due to sample size constraints, no formal consideration of clustering effects at the school level, and the relatively small sample size for subgroup analyses. While we used robust standard errors to address some concerns about correlated observations, future studies could benefit from mixed-effects modeling for more detailed within-person comparisons. In our analysis, combining neutral and negative responses was done to focus on identifying clearly positive responses (scores 4–5), following established practice in tobacco control PSA research. Future studies should examine the full response spectrum.

Future research should adopt longitudinal designs to track adolescent attitude and behavior changes before and after advertisement exposure, expand samples to more cities and rural areas, and consider using more objective physiological measurement indicators to supplement self-reported data.

## CONCLUSIONS

This study evaluated the effectiveness of eight tobacco control PSAs among Chinese adolescents using mixed methods, finding that advertisements employing real-life testimonials, disease warnings, and fear appeals were most effective, while celebrity endorsements and appearance-focused advertisements were least effective. The triangulation of quantitative effectiveness ratings with qualitative explanatory insights provided robust validation of these findings. Regional difference analysis revealed the important influence of tobacco control policy implementation and cultural backgrounds in different Chinese regions on adolescent attitudes.

The findings contribute to understanding culturally appropriate youth tobacco control communication strategies. It is recommended that tobacco control PSA design should adopt strategies combining disease warnings with real-life testimonials, avoid humorous types and industry-sponsored advertisements, and consider regional cultural differences for differentiated communication. With the proliferation of digital media, priority should be given to distribution through online and social media platforms frequently used by adolescents to enhance reach effectiveness and communication impact.

## Data Availability

The data supporting this research are available from the authors on reasonable request.
